# The relationship between Iranian women’s perception of their birth team’s compliance with medical ethics and their perception of labor pain

**DOI:** 10.1186/s12884-024-06269-6

**Published:** 2024-01-20

**Authors:** Parvin Yadollahi, Leila Bozorgian, Roksana Janghorban

**Affiliations:** 1 Department of Midwifery, School of Nursing and Midwifery, Shiraz University of Medical Sciences, Shiraz, Iran; 2 Department of Midwifery, School of Nursing and Midwifery, Shiraz University of Medical Sciences, Shiraz, Iran; 3 Department of Midwifery, School of Nursing and Midwifery, Maternal-Fetal Medicine Research Center, Shiraz University of Medical Sciences, Shiraz, Iran

**Keywords:** Medical Ethics, Labor Pain, Team Birth, Parturient women

## Abstract

**Background:**

A safe and satisfactory childbirth experience with the least amount of pain constitutes one of the main domains of reproductive healthcare. The most important aspect of labor pain management is the moral and professional commitment of the health professionals and caregivers involved in creating a pleasant delivery. The present study examines the relationship between Iranian women’s perceptions of their birth team’s compliance with medical ethics and their perception of labor pain.

**Methods:**

This cross-sectional study was conducted on 200 women opting for natural childbirth. The samples were selected by convenience sampling. Three questionnaires, including a demographic information questionnaire, the perception of labor pain questionnaire, and the medical ethics attitude in vaginal delivery questionnaire, were used to collect data. The data were entered into SPSS 22 and analyzed using correlation coefficient and multiple regression tests. The significance level for data analysis was set as less than 0.05.

**Results:**

The results of the regression analysis showed that among the four principles of medical ethics, only the second and third principles (beneficence and non-maleficence) predicted the perception of labor pain (B = -0.267, *P* < 0.037). Among the different domains of these principles, the areas of giving the necessary information to the mother (B = -0.199, *P* = 0.001), respecting the mother’s privacy (B = -0.194, *P* = 0.001), interaction with the mother (B = -0.287, *P* = 0.001) and assurance of fetal health (B = -0.492, *P* = 0.001) were predictors of labor pain perception score.

**Conclusions:**

Compliance of the birth team with respecting the mother’s privacy, having friendly interactions with the mother and giving fetal health assurance to the mother can be a predictor of the mother’s decreased perception of labor pain.

## Background

Respecting the patients’ dignity and rights is crucial to their satisfaction with the services provided by the healthcare system [1]. There are many concerns regarding compliance with medical ethics in providing services throughout the world, and codes of medical ethics are therefore established based on certain ethical values and beliefs, moral duties and commitments, and cultural characteristics [2, 3]. Healthcare providers and medical specialists are required to provide high-quality health services to patients in a respectful manner by applying the principles of medical ethics [4]. In 1996, the International Confederation of Midwives developed ethical codes for the birth team to promote the health of women and children. In 2014, in efforts to improve the quality of midwifery services across the country, the Iranian Ministry of Health and Medical Education prepared some codes of ethics for birth teams based on the particular work conditions and culture of Iran [3, 5].

One of the key aspects of reproductive health services is providing a safe and pleasant delivery with the least experience of pain. The experience of pain can exert negative physical, psychological and social impact on the patient’s life [6]; the moral commitment of the health team to reducing or eliminating pain and making the experience of childbirth pleasant is a critical aspect of pain management that seems to be significantly overlooked [7].

Labor pain is a common and inevitable phenomenon [8]. For women to reach a positive experience of labor pain, the standard medical treatment approach is simply not enough for reducing the pain experienced; rather, the birth team members must also understand the meaning of pain and recognize the behavioral reactions of women in labor to pain. Birth teams and healthcare providers have to establish proper communication with the parturient, offer emotional support and empathy, teach appropriate coping strategies for pain, avoid negative reactions to the parturient, such as yelling at her or disregarding her needs during labor, and generally show supportive behaviors [9]. Any poor behaviors displayed by the birth team during labor not only increase the parturient women’s fear and anxiety and negative perception of labor pain (PLP), but also sometimes deprive them of receiving professional healthcare services [10]. Therefore, moral performance is one of the main components of maternal and fetal care, and it is necessary for birth team members, especially midwives and obstetricians, to use principles and values ​​that manifest their ethical commitment to the parturient when providing childbirth services [11].

In a study conducted on pregnant women’s fear of childbirth, researchers found the complete lack of trust in the maternity ward staff as one of the major reasons for this fear [12]. A study in Iran revealed that midwives’ compliance with the codes of medical ethics and their respect for the patients’ dignity and privacy are effective in establishing a proper relationship between the parturient and the birth team and reduce the former’s fear and anxiety about the delivery process [13]. Other studies have also found a link between compliance with the codes of medical ethics and the patients’ well-being in different medical fields [10, 14]. Previous research has also shown that the birth team’s compliance with medical ethics has pleasurable consequences for the parturient [15, 16]. Based on the existing evidence, no studies have yet been conducted with a focus on parturient women’s perceptions of the birth team’s compliance with medical ethics and labor pain. The present study was therefore carried out to investigate the relationship between Iranian women’s perception of their birth team’s compliance with the principles of medical ethics and their perception of labor pain.

## Materials and methods

This cross-sectional analytical study was conducted on a sample of 200 pregnant women admitted to maternity hospitals affiliated with Shiraz University of Medical Sciences for natural childbirth. The subjects were collected by convenience sampling from May to July 2020. The inclusion criteria consisted of willingness to participate in the study, being of Iranian nationality, term pregnancy and lack of medical or mental illnesses, wanted pregnancy, no postpartum complications, reading and writing literacy, natural childbirth in a maternity hospital without the use of tools such as forceps and vacuum, and delivery by a midwife or obstetrician. The exclusion criteria were incomplete returning of the questionnaires and unwillingness to participate in the study after filling out the questionnaires. Informed written consent was obtained from all the parturient women 24–48 h after delivery. Then, they completed three questionnaires, including a demographic information questionnaire, the perception of labor pain questionnaire (PLPQ), and the medical ethics attitude in vaginal delivery questionnaire (MEAVDQ).

The PLPQ was validated in Iran by Yadollahi et al. with five dimensions and 31 items, including preparation for experiencing labor pain (five items), nature of labor pain experienced (eight items), internal stressors during labor pain (five items), external stressors during labor pain (four items), and transcendence with labor pain (nine items). In this questionnaire, the items were scored based on a 5-point Likert scale, and the ‘completely agree’ response was given a score of 5 and the ‘completely disagree’ response a score of 1. Items 1–5 and 23–31 were scored in reverse [17]. Higher values represent more negative perception of labor pain.

The MEAVDQ was validated in Iran by Mirzaee Rabor et al. with four principles and 59 items [18]. The first principle of medical ethics is observing autonomy (with 19 items and three domains: Providing the necessary information, respecting the mother’s privacy, and interaction with the mother). The second and third principles are beneficence and none-maleficence (with 27 items and seven domains: The significant role of the birth team, ensuring the health of the fetus, mother’s pain, mother’s stress, mother’s health, mother’s need for pain relief, and mother’s relaxation). The fourth principle of medical ethics was the observance of justice (with 13 items and three domains: Trust in the birth team, the necessity to meet the mother’s requests, and giving the same opportunities to each mother). The answers were arranged on a 5-point Likert scale, from ‘completely agree’ (5 points) to ‘completely disagree’ (1 point). Items 30, 31, 38 and 39 were scored in reverse. A greater score equal to 3.25 for all dimensions was considered as a positive attitude towards birth team’s compliance with medical ethics.

The data were analyzed in SPSS software version 22 (SPSS Inc., Chicago, IL, USA) using the correlation coefficient and multiple regression. *P*-value < 0.05 was considered statistically significant. The research project was approved by the Ethics Committee of Shiraz University of Medical Sciences (IR.SUMS.REC.1398.1286). Informed consent was received from each participant. Confidentiality and anonymity of the participants’ identity was guaranteed. The participants reserved the right to withdraw from the study at any stage.

## Results

The mean age of the participating women was 28.02 ± 6.26 years and most of them were housewives with high school education, primiparous, without a history of abortion, and satisfied with their economic state. Table 1 shows the frequency distribution of the other demographic characteristics and fertility characteristics of the postpartum women.

**Table 1:** Baseline characteristics of the parturient women

Variable	*N* (%)
**Mother’s education**	
Illiterate	2 (1)
Primary	28 (14)
Junior high	50 (25)
High school	88 (44)
University	30 (15)
NS	2 (1)
**Spouse’s education**	
Illiterate	4 (2)
Primary	25 (12.5)
Junior high	46 (23)
High school	84 (42)
University	34 (17)
NS	7 (3.5)
**Mother’s job**	
Housewife	163 (81.5)
Employed	11 (5.5)
Self-employed	16 (8)
NS	10 (5)
**Spouse’s job**	
Homemaker	7 (3.5)
Employed	22 (11)
Self-employed	168 (84)
NS	3 (1.5)
**Satisfaction with the economic status**	
Very unhappy	6 (3)
Unhappy	27 (13.5)
Neither satisfied nor dissatisfied	58 (29)
Satisfied	93 (46.5)
Very satisfied	11 (5.5)
NS	5 (2.5)
**Ethnicity**	
Fars	153 (76.5)
Turk	23 (11.5)
Lur	19 (9.5)
NS	5 (2.5)
**Parity**	
1	75 (37.5)
2	71 (35.5)
3	18 (9)
4	31 (15.5)
5	5 (2.5)
**History of abortion**	
Yes	33 (16.5)
No	144 (72)
NS	23 (11.5)
**Delivery with the help of an accompanying doula**	
Yes	40 (20)
No	127 (63.5)
NS	33 (16.5)

The lowest mean score of medical ethics compliance pertained to the second and third principles (beneficence and non-maleficence), which was 4.03 ± 0.65, and the highest mean score belonged to the fourth principle of medical ethics (justice), which was 4.15 ± 0.71 (Table 2).

**Table 2:** The mean score of the birth team’s compliance with the principles of medical ethics during labor from the parturient women’s perspective

Variable		**Mean ± SD**	
The first principle of medical ethics (observance of autonomy)		4.10 ± 0.58	
Domains	Providing the necessary information	4.09 ± 0.6	
	Respecting the mother’s privacy	4.10 ± 0.76	
	Interaction with the mother	4.12 ± 0.73	
The second and third principles of medical ethics (beneficence and non-maleficence)		4.03 ± 0.65	
Domains	The important role of the birth team	4.04 ± 0.67	
	Ensuring the health of the fetus	4.19 ± 0.8	
	Mother’s pain	3.95 ± 0.91	
	Mother’s stress	4.06 ± 0.8	
	Mother’s health	3.92 ± 0.93	
	Mother’s need for pain relief	3.85 ± 0.85	
	Mother’s relaxation	4.17 ± 0.88	
The fourth principle of medical ethics (justice)		4.15 ± 0.71	
Domains	Trust in the birth team	4.15 ± 0.80	
	The necessity to meet the mother’s requests	4.15 ± 0.87	
	Giving the same opportunities to each mother	4.14 ± 0.77	
Total score		4.08 ± 0.59	

The lowest mean score of labor pain perception pertained to external stressors during labor, which was 3.65 ± 1.05, and the highest mean score belonged to the nature of labor pain experienced, which was 3.83 ± 0.84 (Table 3).

**Table 3:** Mean score of labor pain perception from the parturient women’s perspective during labor

Variable	Mean ± SD
Preparation for experiencing labor pain	3.74 ± 0.91
The nature of the labor pain experienced	3.83 ± 0.84
Internal stressors during labor pain	3.82 ± 0.89
External stressors during labor pain	3.65 ± 1.05
Excellence achieved through labor pain	3.77 ± 0.84
Pain perception	3.77 ± 0.72

A regression analysis was used to predict the score of labor pain perception in the postpartum women based on the mean score obtained for the birth team’s compliance with medical ethics. The findings showed that only the second and third principles of medical ethics (beneficence and non-maleficence) were negative predictors of PLP (B= -0.267, *P* = 0.037). These two principles together accounted for 7% of the total variance in PLP score (Table 4).

**Table 4:** Results of the regression analysis between the principles of medical ethics and labor pain perception

Variable	**Coefficient**	**SD**	**Beta coefficient**	**T statistic**	***P-value***
The first principle of medical ethics (observance of autonomy)	0.115	0.363	0.038	0.316	0.752
The second and third principles of medical ethics (beneficence and non-maleficence)	-0.380	0.181	-0.267	-2.102	0.037
The fourth principle of medical ethics (justice)	-0.096	0.380	-0.033	-0.253	0.801

A multivariate regression analysis was used to more accurately predict the PLP score in the postpartum women. The results showed that giving the necessary information to the mother (B= -0.199, *P* = 0.001), respecting the mother’s privacy (B= -0.194, *P* = 0.042), interaction with the mother (B= -0.287, *P* = 0.001) and fetal health assurance (B= -0.492, *P* = 0.001) were predictors of the labor pain perception score. These variables together predicted 27.9% of the total variance in the labor pain perception score (Table 5).

**Table 5:** Results of the multivariate regression between the principles of medical ethics and labor pain perception

Variable	**Coefficient**	**SD**	**Beta**	**T**	***P-value***
Providing the necessary information	104.584	4.608	-0.199	22.698	0.001
Respecting the mother’s privacy	2.948	1.440	-0.194	2.047	0.042
Interaction with the mother	3.623	1.110	-0.287	3.265	0.001
The important role of the birth team	-2.019	1.191	-0.168	-1.694	0.092
Ensuring the health of the fetus	-6.727	1.515	-0.492	-4.441	0.001
Mother’s pain	-1.295	1.316	-0.113	-0.984	0.326
Mother’s stress	0.597	1.127	0.059	0.530	0.597
Mother’s health	0.816	1.268	0.071	0.644	0.521
Mother’s need for pain relief	1.256	1.127	0.128	1.114	0.267
Mother’s relaxation	-0.517	1.132	-0.048	-0.457	0.648
Trust in the birth team	-2.022	1.139	-0.194	-1.775	0.078
The necessity to meet the mother’s requests	-1.675	1.220	-0.147	-1.373	0.171
Giving the same opportunities to each mother	-0.392	1.088	-0.037	-0.360	0.719

## Discussion

According to the women’s perception, the mean score obtained for the fourth principle of medical ethics (justice) was higher than that of the other principles. This finding was also confirmed among Swedish women, who described their perceptions of a very positive birth experience six to seven years after their delivery. They stated that a trustful and respectful relationship with the midwife was an external factor that formed positive experiences of childbirth [19]. In addition to pregnant women, healthcare providers have also insisted on the importance of the principle of justice. A qualitative study in Iran showed that according to professional healthcare providers, “comprehensive attentiveness” is a concept associated with dignity for pregnant women in the delivery room. Attentive care includes meeting the mothers’ needs, avoiding any type of discrimination in their care, and treating all parturient women fairly based on their needs [20]. Despite the importance of treating pregnant women fairly, some evidence was indicative of discrimination against them. An online cross-sectional survey was conducted in the United States to assess seven dimensions of mistreatment in maternity care based on the world health organization guidelines. These dimensions included physical abuse, sexual abuse, verbal abuse, stigma and discrimination, failure to meet professional standards of care, poor relationship between the mothers and healthcare providers, and health system constraints. The findings showed that one in six women experienced one or more types of mistreatment, and women with low socioeconomic status and of African ethnicity reported more mistreatments. The results of the survey thus showed that discrimination continues to exist in healthcare systems [21].

In the present study, the parturient women gave the lowest and highest mean scores to external stressors during labor and the nature of labor pain, respectively. A study on women’s perception of stressors in the delivery ward showed that healthcare staff-related factors and environmental and equipment-related factors were external factors that induced stress in the labor and delivery ward [22]. In the current study, the women who gave the highest score to the birth team’s compliance with the fourth ethical principle, which consisted of a trustful relationship between the parturient women and the birth team and attentive care, had a lower external stressors’ score in comparison with the other domains of the perception of labor pain questionnaire, such as preparation for experiencing labor pain, the nature of the labor pain experienced, internal stressors, excellence achieved through labor pain, and the perception of pain. Women’s state of mind forms their perceptions of labor pain. Unawareness about labor pain, lack of preparation for experiencing labor pain, and internal stressors such as being concerned about the inability to endure labor pain lead to anxiety about the process of childbirth. Most of the times, the parturient woman is engaged in thoughts of the nature of pain and is catastrophizing labor pain for herself [23].

The main finding of the present study was that only the second and third principles (beneficence and non-maleficence) predict PLP. In other words, if birth teams try to maximize the benefits and minimize the harms of delivery, mothers’ perception of labor pain can be reduced. Studies have shown that decisions contrary to these principles could negatively affect the mothers’ experience of labor and pain, their relationship to the birth team, and even their bonding with their child [24–26].

The domains of medical ethics that could predict the PLP score in the present study included providing the necessary information, observing the mother’s privacy, interaction with the mother and ensuring the health of the fetus. A qualitative study of Iranian parturients showed that the fulfillment of the mothers’ informational needs, including familiarity with the labor ward environment, labor process, care plan and medical procedures, and basic practical care for themselves and their baby, can help provide a sense of control and empowerment in childbirth. Women who are more empowered to confront labor also perceive less pain during this time [27]. Therefore, obstetrical care based on the mothers’ care needs is not merely the provision of care according to ethical principles, but also an approach to help diminish labor pain.

In the present study, respecting the mother’s privacy was one of the main elements in predicting reduced PLP. In agreement with this study, Namujju et al. showed that the lack of respect for the mother’s privacy is one of the main components in the formation of an unpleasant experience of childbirth [28]. Privacy consists of the mother’s feeling toward her identity, dignity, independence, and personal space. Ensuring the mother’s privacy before, during, and after delivery has a critical role in forming a pleasurable childbirth experience and increasing the mother’s satisfaction [29].

Another predictor of the PLP score in this study was interaction with the mother. A trusting and good relationship between the birth team and the parturient woman leads to a positive birth experience and can help manage labor pain more than any other pain relief intervention [30]. According to the model of trust within the mother-midwife relationship proposed by Mirzaee et al., mothers seek “trust in the midwife” through “effective interaction”, “trying to access the healthcare providers”, and “playing an active role during childbirth” [26]. It seems that this model is not limited to midwives and can apply to all members of the birth team.

This study also showed that if mothers are assured about the health of their fetus, they perceive less pain during labor. One of the biggest concerns of any parturient is their infant’s health, and this concern can lead to stress, anxiety, and even fear of childbirth [31]. Studies have demonstrated that mothers’ perceived stress during labor causes pain magnification, and pain magnification alongside rumination and helplessness comprise the three dimensions of pain catastrophizing, which is a phenomenon that draws the individual’s attention to pain stimuli and is associated with worse outcomes [32, 33]. The mother’s tendency to catastrophize pain is a predictor of her labor pain experience. It seems that the stress associated with the infant’s health can affect pain perception during labor by changing the mother’s attention to pain stimuli and her tendency to catastrophize pain [23].

The strengths of this study include examining the perception of labor pain from the perspective of parturient women based on the birth teams’ compliance with the codes of medical ethics. This study was performed in governmental hospitals in Iran and the results cannot be generalized to women who delivered in private hospitals or women in other countries.

## Conclusion

This study showed that the compliance of the birth team with respecting the mother’s privacy, having friendly interactions with the mother and giving fetal health assurance to the mother can be a predictor of the mothers’ reduced perception of labor pain.

## Data Availability

All data generated or analysed during this study are included in this published article.

## Abbreviations

MEAVDQ: Medical Ethics Attitude in Vaginal Delivery Questionnaire
PLP: Perception of Labor Pain
PLPQ: Perception of Labor Pain Questionnaire
